# Supplementary figures and images for: Maximum vertical height during wing flapping of laying hens captured with a depth camera
Source: PLoS One. 2025 Mar 27;20(3):e0312656. doi: 10.1371/journal.pone.0312656 (PMC11949347; doi:10.1371/journal.pone.0312656)

**Fig SM2. Spearman rank correlations between physical measurements taken from hens.**
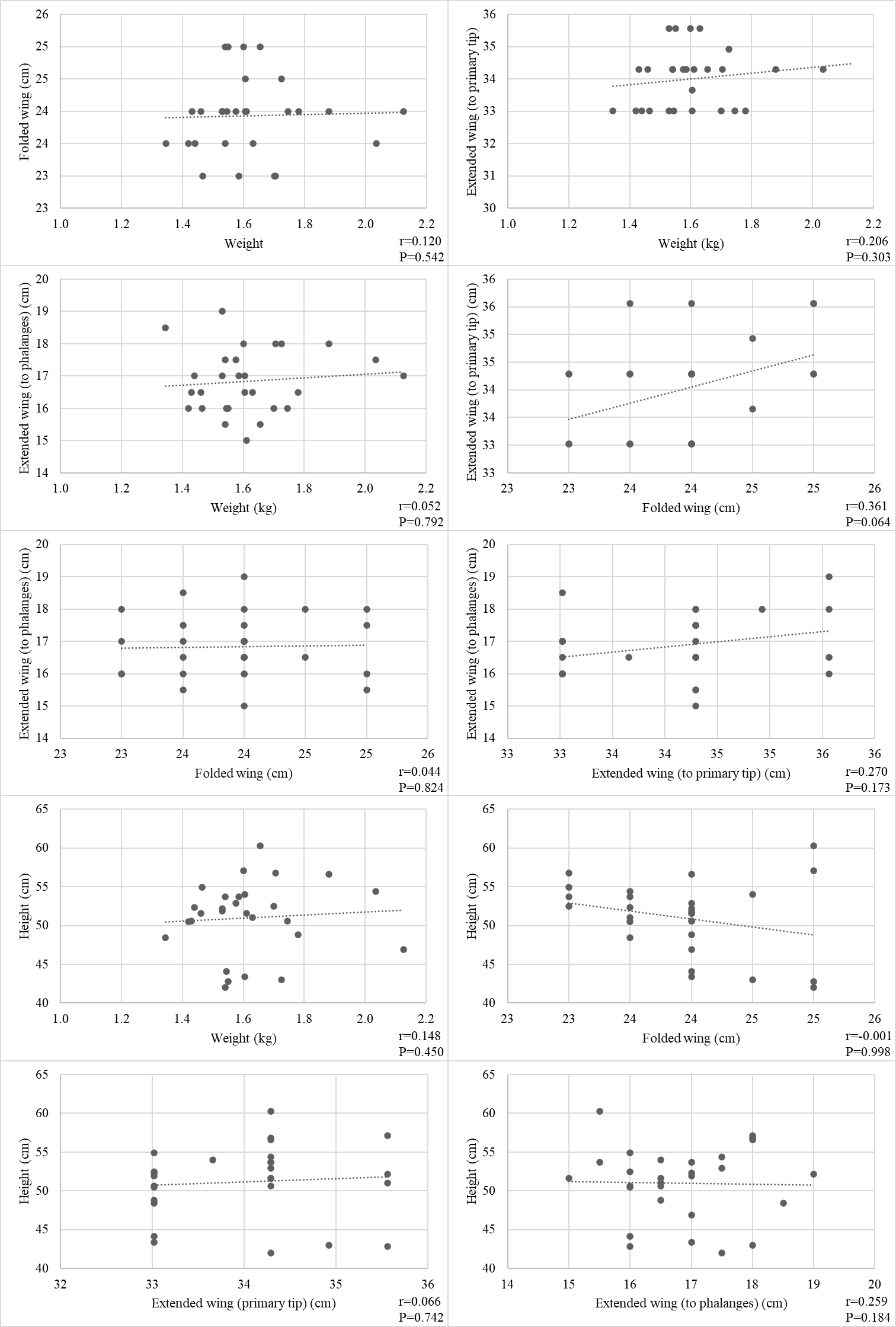

Supplement: S2 Fig — Plots showing Spearman rank correlations between physical measurements taken from hens. (DOCX) [file pone.0312656.s002.docx]
